# Trichloroacetic Acid Peeling for Treating Photoaging: A Systematic Review

**DOI:** 10.1155/2021/3085670

**Published:** 2021-08-30

**Authors:** Irma Bernadette S. Sitohang, Lili Legiawati, Lis Surachmiati Suseno, Fadhila Dea Safira

**Affiliations:** ^1^Division of Cosmetic Dermatology, Department of Dermatology and Venereology, Faculty of Medicine, Universitas Indonesia, Dr. Cipto Mangunkusumo Hospital, Jakarta, Indonesia; ^2^Faculty of Medicine, Universitas Indonesia, Dr. Cipto Mangunkusumo Hospital, Jakarta, Indonesia

## Abstract

Photoaging can significantly contribute to lower quality of life. Medium-deep peeling using trichloroacetic acid allows controlled keratocoagulation through the dermis and into the dermal papillary layer that is effective for skin rejuvenation. The purpose of this article is to give updates regarding the efficacy, possible adverse events, and patient satisfaction of trichloroacetic acid (TCA) peeling for skin rejuvenation by assessing various photoaging parameters. A systematic review of prospective trial articles collected from PubMed, MEDLINE, EMBASE, Cochrane, and Scopus databases was conducted on November 2, 2020. Treatment efficacy was assessed based on the photoaging parameters used by each study. Adverse events and patient satisfaction as the secondary outcome were assessed based on patients-perceived improvements. Five studies included three randomized comparison studies and two prospective cohort studies. These studies show that TCA peeling significantly improve the cosmesis of photoaged facial skin. Low concentration is effective for superficial sun damage. Medium-depth peels using a higher concentration of TCA or as combination therapy are effective as skin resurfacing agents to reduce wrinkles. Some adverse effects may occur but usually resolve within weeks. Overall patients were satisfied with the treatment result. An equivalent basic skin preparation such as topical retinoic acid skin priming prior to intervention is necessary for more objective comparison. Further research studies with a larger sample size and longer follow-up period are required. This evidence suggests that TCA peeling is effective in photoaging treatment, either as monotherapy or as combination therapy with other modalities.

## 1. Introduction

Photoaging is a series of changes in the skin caused by exposure to ultraviolet rays over time. This process overlaps the aging process chronologically, thus making the aging process happen prematurely. In general, several factors can encourage a person to pay more attention and willing to improve the skin appearance, such as social factors, culture, and personal values. To date, many patients choose to use antiphotoaging products or seek professional help for corrective procedures [1]. The skin aging process is a complex series of events consisting of intrinsic or chronological aging and extrinsic aging related to lifestyle and environmental conditions. The intrinsic aging process is greatly influenced by personal skin type, genetic predisposition, and ethnicity. Photoprotective practices such as using sunscreen and finding a shady place to stay can help reduce this process [2, 3].

Although it is not directly related to the mortality rates, photoaging can significantly contribute to higher morbidity with lower levels of quality of life. Therefore, research studies on skin rejuvenation are rapidly being developed by researchers all over the world to seek new modalities that can provide more effective results. One of the most frequently used therapeutic options is chemical peels using a variety of chemical agents. Chemical peels, or chemoexfoliation, can improve skin appearance by causing histological changes in the epidermal level by restoring the condition of atrophy and atypia accompanied by new subepidermal collagen deposition [4]. In patients with extensive actinic keratosis and diffuse photodamage, peeling is preferable for individual lesions [5].

The controlled keratocoagulation process can reach through the dermis and dermal papillary layer in medium-depth peels. Later, this is where the pathological lesions are targeted, resulting in deeper regenerative changes in the epidermis and superficial dermis that can be carried out simultaneously [6]. One of the most commonly used medium peeling agents is trichloroacetic acid (TCA) with a concentration between 30 and 50% [7]. TCA is a chemical cautery that causes protein denaturation which results in a white frost-like appearance called keratocoagulation. TCA peels provide several benefits for both the patient and the doctor. TCA is a solution that is affordable, is easy to prepare, is stable, has a long shelf life, and has no systemic toxicity effect, which is why it is often used as a moderate-deep chemical peels agent for skin rejuvenation purposes [8]. As the development of studies on photoaging treatment advances, efficacy and safety of TCA peeling are increasingly being evaluated clinically in direct comparison with other therapies, both the well-established and the emerging modalities.

## 2. Materials and Methods

The methodology and reporting of this study are based on the guidelines for Preferred Reporting Items for Systematic Reviews and Meta-analyzes (PRISMA) [9]. We had funding sources from Universitas Indonesia. Studies included in this review are prospective clinical trials with patients who received chemical peeling with trichloroacetic acid and other modalities. Animal studies and single case reports were excluded. This review includes articles written in English.

**Figure 1:**
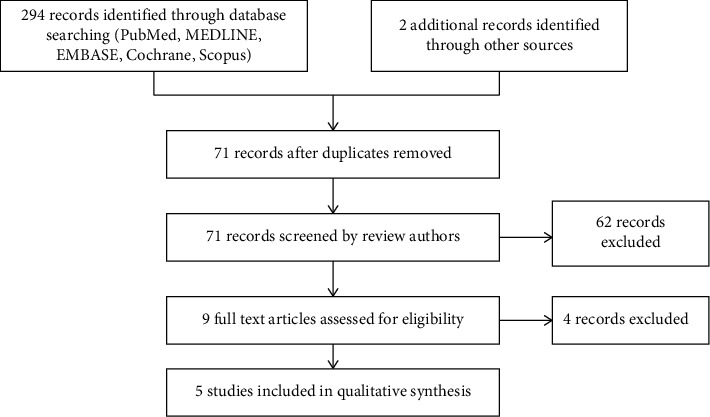
Flow diagram.

The literature search was performed on 25 October 2020, with the databases PubMed, Medline, Embase, Cochrane, and Scopus. Articles used in this review are published within the last 20 years, from January 1, 2000 until October 8, 2020. The MeSH terms used for article searching are “photoag ^*∗*^” OR “wrinkl ^*∗*^” OR “rejuv ^*∗*^” AND “trichloroacetic acid” OR “TCA” AND “peel ^*∗*^.” References within searched articles were also reviewed as additional records for a more comprehensive search (Figure 1).

The articles used were first screened based on their title and abstract by F. D. Safira. Irrelevant articles based on the title and abstract were excluded. We also excluded articles without a full-text version of the manuscript available. Then, articles deemed relevant to the topic will be independently reviewed in full-text by I. B. S. Sitohang, L. Legiawati, L. S. Suseno, and F. D. Safira. If the same datasets are explained in other articles, the reviewer will collect the data from the article with the largest number of patients. The concentration of TCA peeling used in these literature studies varies from 15% to 35% that resulted in superficial to medium-depth peeling that is beneficial as singular or combination therapy for treating photoaged skin.

F. D. Safira independently sorts data from the selected studies into a table. The table elucidates publication details consisted of the title, year of publication, and study design; total patients along with the criteria; details of the TCA peeling intervention; other modalities that are used as the comparison; measurement tools such as scoring or questionnaire; duration of treatment and observation or follow-up period; cosmesis improvement as the primary outcome; patients satisfaction and recorded adverse events as the secondary outcome; and conclusion of the study. The included studies used the Glogau scale for the initial assessment of photodamage degree in patients. Therapeutic outcome was assessed objectively using various photoaging parameters for treatment efficacy and documenting complications. Subjectively, assessments of patients' satisfaction and quality of life were done. The results from the table were then reviewed by I. B. S. Sitohang, L. Legiawati, and L. S. Suseno. The results assessed in these studies were the clinical photoaging parameters used in each study, such as skin elasticity, melanin index, and erythema index. The secondary outcome that was also studied in this review is subject-perceived improvements to assess patient satisfaction with therapy.

## 3. Results

The selected studies included five prospective clinical trial studies comparing various skin rejuvenation modalities with various concentrations of TCA chemical peeling ranging from 15 to 35%. Trichloroacetic acid skin peeling has been commonly used for treating various skin disorders, especially those caused by photoaging such as actinic keratosis and other dermatoses. The depth of the peel varies depending on the way the skin is prepared, acid concentration, and application duration [10]. Its lack of systemic toxicity effects makes TCA peeling a superior therapeutic option for photoaging, and it has been used for a long time [11]. This review included 210 patients in total. Three studies are randomized clinical trials with a total of 145 patients, and two other studies are prospective cohort studies consisting of 65 patients (Supplementary Table 1).

Procedure efficacy in treating photoaging was evaluated with different tools in each study. Yildirim et al. [12] assess the efficacy of TCA peeling using a quartile scale that was graded by three different dermatologists using special computer software designed and developed for the evaluation. Artzy et al. [13] assess the degree of improvement using the global aesthetic improvement scale (GAIS) in four parameters: pigmentation and dyschromia; degree of erythema and blood vessels appearance; laxity and wrinkling; and skin imperfections. Kubiak et al. [14] assess the treatment efficacy by measuring epidermal skin elasticity using Cutometer SEM 474, hydration using Corneometer CM 820, melanin and erythema index using Mexameter MX 18, and depth and volume of wrinkles using PRIMOS. Kubiak et al. [15] assess the treatment efficacy by measuring epidermal skin elasticity using Cutometer SEM 474, hydration using Corneometer CM 820, melanin and erythema index using Mexameter MX 18, and Visioscan 98 for overall skin surface improvements. Holzer et al. [16] assess the treatment efficacy by measuring total lesion count, complete clearance rate of lesion, PGA (physician global assessment) of the target area on a 7-point scale, and new lesion count within the target area using Fotofinder software.

Adverse events that may occur in the TCA peeling procedure can represent as acute or chronic manifestations. Burning sensation and irritation can occur immediately after the procedure is done. In the long term, the most common side effects are hyperpigmentation, hypopigmentation, and scar formation. Despite being the most commonly employed method for a long time, TCA peeling must be monitored carefully and done appropriately. Side effects can also appear in unfavorable postprocedure conditions, such as early exposure or nonprotection from the sunlight.

### 3.1. Cosmesis Improvement

All the included studies show clinical improvement of photoaging after treatment with various concentrations of TCA peeling. However, some of the studies show that other modalities can be as effective as TCA peeling or there are additional modalities that can increase the efficacy of TCA peeling. In this study, it was also found that the application of TCA as a pretreatment for other interventions can reduce the treatment intervention due to changes in skin impedance that it causes.

In 2016, Yildirim et al. [12] conducted a study with 50 photodamaged patients and formed two randomized treatment groups: TCA 25% chemical peeling and 0.1% retinoic acid topical treatment for four months. At the end of the treatment, all observers discover no significant clinical efficacy difference in both treatment groups. At the last follow-up, three months after the last treatment, two observers reported patients subjected to retinoic acid had statistically significant higher healing rate in the final follow-up visit, while another observer reported a similar result with no statistically significant difference.

In 2019, Artzy [13] et al. prospectively compared four protocols for 67 patients that were assigned randomly to four different centers. Center A provided the microneedling bipolar fractional radiofrequency (FRF) technology, Center B provided 20% TCA chemical peel followed immediately by FRF treatment, Center C provided FRF treatment followed by chemical peeling using 20% TCA, and Center D provided a monotherapy of 20% TCA chemical peel. The patients were given 3–5 treatments with 4–6-week interval. The FRF technology gave thermal damage that is localized and focal to the tissue by inducing a regenerative mechanism. Improvements in the skin imperfections and overall skin condition pretreatment and posttreatment scores were found in all four groups. The group of patients who showed the most preferable results using the global aesthetic improvement scale (GAIS) at six months after the last treatment evaluation was the group who received FRF treatment followed immediately by 20% TCA2, especially in laxity and wrinkling, as well as pigmentation and dyschromia (*p* < 0.001). Redness and other skin imperfections were showing no significant improvement. This study showed a reduction in skin impedance with the application of 20% TCA before FRF. The application of TCA peeling made the energy from FRF therapy not being used efficiently because microneedling penetration became more superficial.

Kubiak et al. [14] conducted a prospective comparison study of 20 type II and III Glogau photoaging scape patients. The patients were assigned to two groups, 15% trichloroacetic acid peel with 70% glycolic acid (GA/TCA) and 35% trichloroacetic acid peel (TCA) for five sessions with 14-week interval. Significant improvement in both arms was found in the assessment of skin elasticity, hydration status, and melanin and erythema index. Hydration and elasticity parameters were shown to present a better improvement in proportion to the number of sessions given. GA/TCA group demonstrated significantly better skin hydration. About 35% TCA peel demonstrated a significantly higher melanin index and reduction of wrinkle depth than GA/TCA combination therapy.

Kubiak et al. [15] conducted a prospective comparison study between 70% glycolic acid (GA) and 15% TCA for chemical peeling in treating photoaging in 10 weeks with 14 days of interval between each session. Significant improvements using both GA and TCA peels were observed as early as the 4th week of the study. All patients were satisfied with the treatment. There was no significant difference between TCA and GA groups in improvement of skin elasticity. Improvement of moisture was observed in both groups after 20 weeks with a significant statistical difference stating GA is more superior than TCA. Melanin intensity decreased significantly after GA peeling but was not statistically significant with TCA peeling. An increase of erythema was observed in patients treated with both TCA and GA peeling within the first five treatments. After 20 weeks, reduction of erythema was observed to be statistically significant only in the TCA group. Surface evaluation using Visioscan reported improvement of skin surface with no significant difference between each group.

Holzer et al. [16] conducted a randomized, observer-blinded, intrapatient comparison study of 35% TCA peeling and 20% aminolevulinic acid photodynamic therapy (ALA PDT) in treating multiple actinic keratosis for 12 months. Significant cosmetic improvement was found in both of the groups. ALA PDT showed better clinical appearance than TCA in reducing total lesion count, complete clearance of preexisting actinic keratosis lesion, and decreasing rate of treatment failure. Even so, both of the groups did not differ significantly in the global cosmetic improvement assessment at the end of the study.

### 3.2. Adverse Events

Common adverse events of acid-base chemical peeling were stinging and burning sensation. There was mild erythema that only lasted for a few minutes after treatment. Yildirim et al. [12] found that burning and irritation complaint was recorded to be more severe in patients receiving TCA peeling than in patients treated with topical retinoic acid. However, another study conducted by Kubiak et al. [15] reported that the discomfort and stinging on the use of 15% TCA peel was lighter than that by 70% GA peels. Burning and stinging sensation on the 15% TCA peel treatment was felt immediately and most pronounced at the first visit but improved on subsequent visits. Holzer et al. [16] reported treatment-related pain was present in 35% TCA peeling treatment but was significantly lower than in ALA PDT treatment.

Kubiak et al. [14] reported that the incidence of erythema caused by peeling increased with a higher concentration of TCA solution. Addition of other chemical agents such as glycolic acid did not increase the risk of erythema after peeling. However, the risk of mid-dermal necrosis increased with the addition of 70% glycolic acid prior to the application of TCA. Holzer et al. [16] reported the presence of erythema and scaling after TCA and ALA PDT treatment. These side effects are usually resolved within one week. Even so, persistent erythema was present for up to one month after treatment.

Adverse event of hyperpigmentation was similar in TCA and retinoic acid treatment [12]. However, the formation of scar was not detected in any of the participants, and only one person developed hypopigmentation. Holzer et al. [16] reported scarring was only present in TCA-treated patients. Some of the patients required topical steroids and onion extract gel treatment. One bacterial superinfection case was also reported in this study.

Unlike the case of topical therapy, Arty et al. [13] showed longer downtimes where the patients experienced reduced activity due to discomfort and severity of the erythema, edema, crusting, and blistering was significantly reported in FRF therapy followed by TCA peeling although this combination showed the best result on skin rejuvenation. This combination resulted in deeper TCA penetration. The histological examination demonstrates signs of burn such as thinned, detached, and epidermal necrosis with foci of ulceration in all groups that received FRF treatment. Only the group that received TCA 20% as a monotherapy showed no signs of burn clinically and histologically.

### 3.3. Patient Satisfaction

A significant improvement in the quality of life of patients with photoaging has been observed after TCA peeling treatment [12]. Subjective evaluation conducted by Kubiak et al. [15] showed a remarkably satisfactory response towards the treatment of TCA and GA peeling result. Subjectively, patients felt that TCA treatment is superior in improving skin elasticity and reducing skin wrinkling. Combination therapy of GA/TCA did not differ significantly from 35% TCA peel in subject-perceived improvement [1].

## 4. Discussion

TCA is a superficial chemoexfoliation agent that is popular for treating mild signs of aging and evens out the skin's surface. Superficial peels reduce the stratum corneum thickness by causing protein precipitation and cell coagulative necrosis in the epidermis. It improves the quality of elastic fibers and promotes the synthesis of collagen fibers by increasing the thickness of the mucopolysaccharide of the ground substance [14, 15]. TCA properties are commonly used for treating other superficial skin problems such as acne vulgaris of melasma because of its keratolytic property by solubilizing intercellular cement, thus reducing the adhesion of corneocytes [15]. With more than 15% concentrations, TCA reaches the papillary layer to the upper reticular dermis and causes collagen necrosis [14]. Acidic properties of TCA cause protein coagulation and skin frosting, followed by necrosis of keratinocytes and skin exfoliation [17].

Frosting as the target result of TCA peeling resulted from protein coagulation in the epidermal and dermal layers. Superficial injury as the desired target of lower TCA peeling concentration (15% TCA) is marked by the presence of diffuse skin erythema with light cloudy white frosting that clears in 10–15 minutes. This sign of frosting does not always present in a low concentration of TCA peels. Deeper epidermal and papillary dermal injury by a slightly higher concentration of TCA peels (35% TCA) is marked by the presence of a regular white frost and also clears over 10–15 minutes [15]. TCA application longer in the concentration of 35% than 15 seconds must be carefully monitored because it can blanch the skin [18]. Concentration of TCA above 40% creates a deep peel that is difficult to control and may yield scarring and pigmentation disorder [10].

Quality of elastic and collagen fibers is improved due to the promotion of collagen fiber synthesis, as well as increases water and glycosaminoglycan content in the dermis to promote rearrangement of the ground substance [13]. Superficial peels also reduce the stratum corneum thickness while enhancing collagen fiber's synthesis to delay the photoaging process and improve the appearance of sun-damaged skin [18, 19]. A significant increase in thickness of the dermal layer and glycosaminoglycan content was found in patients treated with TCA peeling due to improvement in quantity of dermal collagen and elastic fiber rearrangement [20].

Medium-depth peeling can be achieved by a combination of chemical peeling or higher concentration. TCA peeling in the concentration of 35% was proven to be effective in reducing wrinkling depth significantly. Combination of GA/TCA and 35% TCA were notably effective in the removal of actinic keratosis and other mild photoaging signs such as rhytides, pigmentary dyschromia, and textured skin [14]. Therefore, superficial TCA peels are more frequently applied for skin refreshment, while medium-depth TCA peels with a concentration of 35% or combination with other agents in lower concentration are effective as a resurfacing skin agent [17]. TCA as a chemical peeling agent appears as a promising modality for epidermal signs of photoaging such as actinic keratosis because it treats large skin surface areas in a single session and may improve the appearance of chronically photodamaged skin simultaneously [16]. This is a cost-effective method with excellent cosmetic results [15].

The patients will experience discomfort such as stinging and burning sensation during application. This discomfort usually subsided by the end of the appointment [21]. A series of low-concentrated TCA peels are proven to be well tolerated by patients [15]. Skin priming with topical retinoic acid may prepare the skin before receiving the treatment. None of the studies reported the usage of skin priming prior to treatment application. Hyperpigmentation associated with unprotected sunlight exposure is one of the most frequent complications following chemical peeling, especially with TCA [18]. None of the studies included in this review demonstrate this adverse effect [12–15].

Combination therapies such as microneedle FRF treatment that creates microwounds prior to the peeling procedure can result in a deeper peel effect because it allows the chemical peeling agent to pass through the transdermal delivery and thereby lead to better results. However, adverse effects due to deeper penetration need to be considered [13]. On the other hand, there are other cost‐efficient options with more or less similar efficacy with independent TCA peeling for photoaging treatment. The topical retinoic acid cream is one of the effective and reliable examples. However, the result relies on the patient compliance [12].

## 5. Conclusions

The development of therapy to prevent and improve the appearance of aging skin due to sun damage has been widely adopted, both invasive and noninvasive methods. Skin peeling with TCA has been significantly proven to be successful in treating photoaging. Despite being one of the most popular chemical peeling agents, appropriate usage must be reassured because of its possible adverse events and complications. Side effects such as burning and stinging sensation right after treatment are noticeable but can easily be controlled [13, 15].

TCA peels significantly improve photodamaged facial skin of patients [12–15]. Improvements in skin conditions such as elasticity and hydration, and signs of photoaging such as hyperpigmentation, fine lines, dryness, and erythema have been shown to improve along with the number of repetitions of the treatment given [14, 15]. Less frequent but continuous use of superficial TCA peeling is beneficial to keep ongoing improvement [15]. Further research is required to ascertain the potential of TCA peeling as a photoaging treatment with a larger sample size and longer follow-up period in comparison with other modalities or as combination therapy to provide cost-effective treatment strategies.

## Data Availability

The review table data used to support the findings of this study are included within the supplementary information file.

## Supplementary Materials

Supplementary Materials Supplementary file 1 is an appraisal of five articles included in this study in the form of a review table. The extracted data included the title, year of publication, study design, number and characteristics of participants, intervention and comparison group description, the measure of outcome variables, duration of treatment and follow-up, cosmesis improvement as the primary outcome, patients satisfaction and recorded adverse events as the secondary outcome, and the conclusion of the study.
