# Supplementary material for: Trichloroacetic Acid Peeling for Treating Photoaging: A Systematic Review
Source: Dermatol Res Pract. 2021 Aug 30;2021:3085670. doi: 10.1155/2021/3085670 (PMC8423570; doi:10.1155/2021/3085670)
Supplement: Supplementary Materials — Supplementary file 1 is an appraisal of five articles included in this study in the form of a review table. The extracted data included the title, year of publication, study design, number and characteristics of participants, intervention and comparison group description, the measure of outcome variables, duration of treatment and follow-up, cosmesis improvement as the primary outcome, patients satisfaction and recorded adverse events as the secondary outcome, and the conclusion of the study. [file 3085670.f1.docx]

## Supplementary Materials

Table 1. Review Table

| **No** | **Title (Year), Author, Study Design** | **Patients (n), Criteria** | **Intervention** | **Comparison** | **Measurement** | **Duration of treatment and follow up** | **Primary Outcome** | **Secondary Outcome** | **Conclusion** |
| --- | --- | --- | --- | --- | --- | --- | --- | --- | --- |
| 1 | Comparison of efficacy of chemical peeling with  25% trichloroacetic acid and 0.1% retinoic acid  for facial rejuvenation ^10^ (2016)  Yildirim S, Gurel MS, Gungor S, Tekeli O, Canat D.  Randomized clinical trial. | Total: 50 patients  Inclusion criteria:   - Female patients age 30-60 years old with medium-advanced photoaging (Glogau II-III) - Fitzpatrick II-IV skin type - Treatment naïve - Could participate in the follow-up visits regularly - Had not presented with active infection and inflammatory dermatosis in the treatment region - Had not had any significant collagen tissue disease, cardiovascular, pulmonary, renal or psychiatric disease history.   Exclusion criteria:   - Using medications that might lead to photosensitization - Patients with photosensitive disease - Having a hypertrophic scar or keloid tendency - Patients undergoing the isotretinoin treatment for the last 6 months - Recent operation in the facial region - Pregnant women and nursing mothers | 25% TCA skin peeling once every month. | 0.1% retinoic acid peeling treatment applied 5 nights of weekdays | - Three dermatologists measure treatment response in quartile range: (0) no response, (1) minimal response, (2) partial response, (3) good response and (4) optimal response. - Likert scale for burning and irritation by the patients immediately post-treatment: none (0), very mild (1), mild (2), severe (3) and very severe (4). - Turkish Dermatology Life Quality Scale questionnaire for social, emotional status, and daily activities as well as sexual life and symptoms of those patients. | Duration: four months.  Follow up: every month for four months and three months after treatment. | Retinoic acid presented a statistically significant higher healing rate compared to TCA according to two observers. One observer declared the same result with no statistical significance. | - Burning and irritation was reported to be more severe in the TCA group - Hyper- or hypopigmentation and scar formation were not statistically significant between both groups. - Both of the groups presented improvement in quality of life. | 25% TCA peeling is as effective as 0.1% retinoic acid peeling for treating photoaging |
| 2 | Radio Peel—Synergism Between Nano-fractional Radiofrequency and 20% Trichloroacetic Acid Chemical Peeling. (2019)  Artzi O, Cohen S, Verner I, et al.  Multicenter randomized prospective clinical comparison study. | Total: 67 patients  Inclusion criteria:   - Healthy subjects age 18-60 years old.   Exclusion criteria:   - Unable or unwilling to follow the treatment protocol - History of poor wound healing - Active lesion in the treated area - History of keloid formation - HIV, hepatitis or immunocompromised condition - Current pregnancy or lactating - Use of oral retinoids in the previous 12 months - History of deep chemical peels or laser resurfacing procedures within the last 6 months | TCA skin peeling with 20% concentration | Microneedling bipolar fractional radiofrequency (FRF) technology, TCA 20% peeling followed by FRF, and FRF followed by TCA peeling | The patients and two dermatologists assess the degree of improvement using the global aesthetic improvement scale (GAIS) in four parameters:   - pigmentation and dyschromia - erythema and blood vessels - laxity and wrinkling - skin imperfections   The patients’ satisfaction was rated on a numerical scale.  Adverse events were recorded by quantify the period of downtime to assess treatment safety.  Skin impedance and histological changes following the different protocols were evaluated on 3 additional volunteers. | 3 to 5 treatments at 4-to 6-week intervals. | - GAIS scores were significantly lower (indicating better cosmesis) for the FRF -> TCA treatment protocol compared with the other 3 protocols in laxity and wrinkling as well as pigmentation and dyschromia. - There were no significant differences in redness and skin imperfections between the 4 protocols | - No complications documented. - FRF 🡪 TCA resulted in longer downtimes - TCA20% group showed no signs of burn clinically and histologically. | FRF🡪TCA yielded the best result on skin rejuvenation but resulted in longer downtimes  . |
| 3 | Comparative study of 15% trichloroacetic acid peel combined with 70% glycolic acid and 35% trichloroacetic acid peel for the treatment of photodamaged facial skin in aging women. (2019)  Kubiak M, Mucha P, Rotszjen H.  Prospective cohort study. | Total: 40 patients  Inclusion criteria:   - Healthy women, aged between 41 and 60 - Type II and III Glogau photoaging scale - Presented benign skin lesion: dryness, wrinkling, pigmentary dyschromia, and erythema.   Exclusion criteria: not explained | 35% trichloroacetic acid peel | 15% trichloroacetic acid peel combined  with 70% glycolic acid | - Epidermal skin elasticity: Cutometer SEM 474 - Hydration: Corneometer CM 820 - Melanin and erythema index: Mexameter MX 18 - Depth and volume of wrinkles: PRIMOS - Subjective evaluation and irritation complaints assessed according to the following scale: 0 = none, 1 = mild, 2 = moderate, 3 = severe. | Treatment duration: five peel sessions with 14 days intervals. (10 weeks)  Follow-up examination: beginning of study, before each treatment, and 3 months after the last application. | - Significant clinical improvement in both groups for all parameters - Elasticity improvement of TCA was slightly greater than GA/TCA - GA/TCA was characterized by significantly higher values of the hydration parameter and lower values of melanin index compared with 35% TCA - Depth of wrinkling reduction was found to be more significant in the TCA group | - Combination peel GA/TCA did not cause dryness, edema, or intensive lysis of the epidermis - The frequency of peel‐induced erythema did not increase with the addition of glycolic acid, but with a higher concentration of the TCA solution. - Subject‐perceived improvements of the 35% TCA peel did not differ significantly from combination peel treatment. - Marked adverse events were not observed in either group | Both medium‐depth chemical  peels proved to  be useful for the removal of epidermal or superficial lesions and to improve  grade II‐III Glogau photoaged skin  35% TCA peel is more effective  in reducing wrinkle  The addition of glycolic acid before 15% TCA chemical peel application significantly enhanced the increase in skin elasticity and hydration; reduction in melanin index and erythema  index. |
| 4 | Evaluation of 70% Glycolic Peels Versus 15% Trichloroacetic Peels for the Treatment of Photodamaged Facial Skin in Aging Women. (2014)  Kubiak M, Mucha P, Debowska R, Rotszjen H.  Prospective cohort study | Total: 25 patients  Inclusion criteria:   - Healthy women aged 41-60 years old with photodamaged skin. - Patients with Fitzpatrick skin Types II-III - Glogau photoaging scale Types II and III - Patients had at least 3 of the 4 signs of photoaging (mild-to-moderate hyperpigmentation as well as fine lines and wrinkles, dryness, and erythema).   Exclusion criteria:   - Patients with dermatologic disorders that would interfere with the test results or increase risks of adverse reaction. | 15%  Trichloroacetic acid Peels | 70% Glycolic Peels | - Epidermal skin elasticity: Noninvasive in vivo suction skin elasticity meter equipped with 2-mm measuring probe (Cutometer SEM 474) - Hydration of the stratum corneum: non-invasive skin capacitance meter   (Corneometer CM 820)   - Melanin and erythema index: narrow-band reflectance spectrophotometer (Mexameter MX 18) - Skin improvements (smoothness, roughness, and wrinkles): video sensor chip with a very high resolution, an objective and UVA light source (Visioscan 98)   Subjective irritation complaints assessed according to the following scale: 0 = none, 1 = mild, 2 = moderate, 3 = severe. | Treatment duration: five peel sessions with 2 weeks interval ( 10 weeks) Follow up: before each treatment  and 3 months after the last application. | - Elasticity improvement was similar in both groups - GA is more superior in TCA in improving skin moisture - Melanin intensity decreased significantly after GA peeling but not statistically significant with TCA peeling - Erythema was increased in both groups for the first 5 weeks but reduce after 20 weeks significantly only in the TCA group - Both groups showed improvement of skin surface with no significant difference. | - Subjective evaluation showed a good or very good response (GA, 84%; TCA, 68%) which was statistically insignificant. - The 70% GA procedure caused more discomfort and strong stinging during the application - 15% TCA peel was associated with immediate stinging and burning that was most pronounced at the first visit | 70% GA and 15% TCA as superficial peels proved to be an effective treatment modality  for photodamaged facial skin.  Glycolic acid increases skin’s hydration faster. |
| 5 | Randomized controlled trial comparing 35% trichloroacetic acid peel and 5‐aminolaevulinic acid photodynamic therapy (ALA PDT) for treating multiple actinic keratosis. (2016)  Holzer G, Pinkowicz A, Radakovic S, Schmidt JB, Tanew A.  Randomized controlled trial | Total: 28  Inclusion criteria: Patients with five actinic keratoses lesions in two comparable anatomical areas on the head  Exclusion criteria:   - Pregnancy - Immunosuppression - Topical treatment within the last two weeks before inclusion into the study - Systemic retinoid therapy within the previous six months - Patients under ongoing therapy with oral anticoagulants - Contraindication against PDT or TCA peeling such as local skin infections or sensitization against 5-ALA or TCA. | 35% trichloroacetic acid peel | 5-aminolaevulinic acid 20% photodynamic therapy | Therapeutic efficacy and cosmetic outcome was assessed by measuring the reduction of the total lesion count, complete clearance of pre-existing actinic keratosis lesions, PGA (physician global assessment) of the target area on a 7-point scale (0=completely cleared, 1=almost clear, 2=mild, 3=mild-to-moderate, 4=moderate, 5=moderate to severe, 6=severe), and new lesion count within the target area using Fotofinder software.  Adverse events were assessed by recording treatment-related pain using the Visual Analogue Scale and scarring. | Treatment duration: 12 months.  Follow up:  Assessments were done by a blinded investigator 1,3,6, and 12 months after treatment. | - Reduction of the total lesion count was found 31% in the TCA group and 58% in the ALA PDT group.  - Complete clearance of pre‐existing lesions was 49% for TCA and 74% for ALA PDT.  - Treatment failure was observed in seven patients (25%) after TCA and in two patients (7%) after PDT treatment. | -Treatment related pain was significantly higher for ALA PDT (VAS 7·5 ± 2·3 vs. TCA: 5·1 ± 2·6)  - Scarring (n = 6, 21%) was seen only in TCA-treated patients. | ALA PDT provided better clinical results than TCA in the treatment of patients with extensive field cancerization and multiple AKs. |
